# Evidence synthesis of postoperative pain with bioceramic vs. epoxy resin sealers: umbrella review of randomized trials within existing systematic reviews

**DOI:** 10.3389/fdmed.2025.1749298

**Published:** 2026-01-16

**Authors:** Mrunali Dahikar, Ashish Mandwe, Kulvinder Singh Banga, Alexander Maniangat Luke, Suraj Arora, Unmesh Khanvilkar, Ajinkya M. Pawar

**Affiliations:** 1 Department of Conservative Dentistry and Endodontics, Nair Hospital Dental College, Affiliated to Maharashtra University of Health Sciences (MUHS), Mumbai, Maharashtra, India; 2 Department of Clinical Science, College of Dentistry, Ajman University, Al-Jurf, United Arab Emirates; 3 Centre of Medical and Bio-Allied Health Sciences Research (CMBAHSR), Ajman University, Al-Jurf, Ajman, United Arab Emirates; 4 Department of Restorative Dental Sciences, College of Dentistry, King Khalid University, Abha, Saudi Arabia; 5 Department of Conservative Dentistry and Endodontics, Bharati Vidyapeeth Dental College, Navi Mumbai, Maharashtra, India

**Keywords:** bioceramic sealer, endodontics, epoxy resin sealer, postoperative pain, single-visit endodontics

## Abstract

**Objective:**

The evidence on postoperative pain and clinical outcomes in patients receiving primary non-surgical root canal therapy with bioceramic vs. resin-based sealers was compiled in this comprehensive review from systematic reviews and meta-analyses.

**Methods:**

The review followed PRISMA 2020 guidelines and was registered in PROSPERO (CRD42023461029). Systematic reviews included randomized or quasi-randomized trials of adult patients having treatment with either sealer type for postoperative pain, and used validated scales. Screening, data extraction, and quality assessment by A MeaSurement Tool to Assess systematic Reviews 2 (AMSTAR-2) were completed independently by two reviewers and review overlap was measured with the Corrected Covered Area (CCA). Where feasible, *de novo* random-effects meta-analyses were conducted to estimate standardized mean differences (SMD) in pain at 24 and 48 h. Heterogeneity was measured using the *I*^2^ statistic and certainty of evidence with GRADE.

**Results:**

Seven reviews (2020–2024) met eligibility, five with quantitative synthesis. Pooled analyses showed no significant differences in pain between sealer types within the first 6–48 h. Detected differences were small and clinically negligible. Both sealers showed similar analgesic use and flare-up rates. Methodological quality ranged from moderate to low; certainty of evidence for early pain was moderate and low for pain at >48 h due to study inconsistency and imprecision.

**Conclusions:**

Bioceramic sealers offer only a minimal, clinically insignificant reduction in early postoperative pain compared to resin-based sealers. Both nevertheless remain suitable options for reducing patient discomfort. Future studies should standardize pain evaluation, include retreatment cases, and explain clinically significant findings.

**Systematic Review Registration:**

https://www.crd.york.ac.uk/PROSPERO/view/CRD42023461029, PROSPERO CRD42023461029.

## 1 Introduction

Even though endodontics achieved a lot of innovation in both technology and methods, post-operative pain continues to be one of the most prevalent and most difficult complications of root canal treatment (1). The use of rotary and reciprocating instruments, activated irrigation and thermoplastic obturation greatly improved the clinical outcomes; still, many patients complain of pain during the initial healing phase. The reported occurrence of post-operative pain following non-surgical root canal treatment (NSRCT) is known to vary from 2.5% to 60%; reported estimates suggest that almost 40% of patients will experience some pain within the first 24 h of treatment, and nearly 10% will remain symptomatic after one week (2, 3). Persistent post-operative discomfort represents both a clinical challenge and a patient-related issue, affecting satisfaction, acceptance of treatment, and overall perceived quality of care.

There are multifactorial reasons for post-operative pain encompassing mechanical, microbial, and chemical events. The biological interaction of root canal sealers with periapical tissue has received particular attention in the literature, and sealer extrusion and/or irritation may elicit a short-lived inflammatory response, establishing pain and irritation to periradicular tissue (4, 5). Chemical irritants, cytotoxicity, ion release, and interactions of the cement with dentin are important contributors to the extent and duration of post-treatment inflammation (6).

Historically, epoxy resin–based sealers such as AH Plus and ADseal have been considered the gold standard due to their favorable sealing capacity, low solubility, and dimensional stability (7). However, their composition—based on bisphenol-A epoxy resins and amine hardeners—has raised biocompatibility concerns. The release of unpolymerized monomers and trace formaldehyde during polymerization may provoke periapical cytotoxic effects and local inflammatory reactions (8). To overcome these limitations, calcium silicate–based or hydraulic bioceramic sealers were introduced as bioactive alternatives. Bioceramic sealers like EndoSequence BC and iRoot SP have a high biocompatibility due to their alkaline pH and continuous release of calcium and silicate ions, and they promote the formation of hydroxyapatite (9–11). Recently, scientists have been studying these bioceramic sealers at a cellular and tissue level. Inada et al. (12), performed a dentin-tube experiment and showed that a ready-made bioceramic sealer exhibited better bioactivity, porosity, and interfacial adaptation compared to epoxy resin-based sealers, all while showing excellent biocompatibility. Similarly, Shokrzadeh et al. (6), showed the bioceramic sealer had improved mineralization potential and less cytotoxicity to gingival fibroblasts compared to AH Plus. While laboratory studies have consistently shown bioceramic sealers showing superior bioactivity than AH Plus, but it is important to note that clinical evidence has not consistently shown that this translates into reduced postoperative pain or periapical inflammation.

Numerous randomized controlled trials and systematic reviews have compared bioceramic and resin-based sealers in terms of postoperative pain, but the conclusions were contradictory. Some clinical trials noted temporary reduction in early pain after using bioceramic sealers, however, most meta-analyses found there were no clinically meaningful differences (13–17). Even when there are statistically significant differences, the standardized mean differences (SMDs) reported for pain response in these reviews (approximately −0.15 to −0.25) were not of clinically meaningful superiority (18). These differences in findings could have arisen due to several methodological differences such as pulp status, study treatment protocols (single vs. multiple visits), irrigation and obturation techniques and methods of pain response assessment. Further, previous meta-analyses have employed different inclusion criteria and statistics, often from overlapping studies, adding to the complexity in the interpretation of these trials.

Due to this variability, there has been an emerging emphasis on higher order evidence synthesis to improve understanding. Umbrella reviews, a systematic review of systematic reviews, provide a broad approach that accounts for overlapping meta-analytic data, quantifies redundancy and assesses quality using tools like AMSTAR-2 (19). Umbrella reviews are specifically useful in endodontics where multiple reviews repeatedly examine the same questions or sub-questions but have minor inconsistencies in eligibility criteria or statistics.

The present ambiguity surrounding the effect of sealer type on postoperative pain does not result from a dearth of research (20, 21). Rather, the literature is inconsistent, conflicting, and fragmented. Although we have several systematic reviews on the topic, differences in confidence levels and methods for conducting analyses have not resulted in a conclusion (22–24). Therefore, clinicians are faced with a large body of literature that is ambiguous and does not offer clear clinical advice about advantageous postoperative outcomes of bioceramic over resin-based sealers in primary NSRCT.

Accordingly, the purpose of this umbrella review is to critically appraise and synthesize the best available evidence comparing bioceramic and resin-based sealers in primary NSRCT. By considering overlap, methodological quality, and whether there is a certainty of findings in systematic reviews and meta-analyses, this umbrella review aims to clarify the ambiguity that exists and provide clinicians with sound, reliable evidence for sealer selection in endodontics with respect to patient-centered postoperative pain.

## 2 Methodology

### 2.1 Protocol and reporting

This comprehensive review complied with the Cochrane Handbook for Systematic Reviews of Interventions' methodological guidelines and the Preferred Reporting Items for Systematic Reviews and Meta-Analyses (PRISMA) 2020 statement. The Joanna Briggs Institute (JBI) Reviewer's Manual for umbrella reviews served as additional guidance for the methodology. The International Prospective Register of Systematic Reviews (PROSPERO; registration ID: CRD42023461029) received a prospectively registered full protocol.

### 2.2 Eligibility criteria

Eligibility criteria were defined using a PICOS framework (Table 1). Systematic reviews and meta-analyses were considered eligible if they included randomized controlled trials (RCTs), quasi-RCTs, or controlled clinical trials comparing bioceramic (hydraulic calcium silicate–based) sealers with epoxy resin–based sealers in adult patients undergoing primary, single-visit, non-surgical root canal treatment (NSRCT).

**Table 1:** PICOS framework of the umbrella review.

PICOS component	Description
Population (P)	Patients undergoing non-surgical root canal treatment of permanent teeth, irrespective of age, sex, or pulp status, as reported in the included reviews
Intervention (I)	Root canal obturation using bioceramic (calcium silicate–based) sealers, including but not limited to EndoSequence BC, iRoot SP, TotalFill BC, and Endoseal MTA
Comparator (C)	Root canal obturation using resin-based sealers, predominantly epoxy resin–based formulations such as AH Plus
Outcomes (O)	Primary outcomes: Postoperative pain intensity assessed at 24 h and 48 husing validated pain scales (e.g., VAS)
Secondary outcomes	Pain beyond 48 h, analgesic consumption, and flare-up incidence
Study design (S)	Systematic reviews and meta-analyses of randomized controlled trials (RCTs) evaluating postoperative pain outcomes following root canal treatment

Eligible reviews were required to report postoperative pain as either a primary or secondary outcome, measured using validated scales such as the Visual Analogue Scale (VAS) or Numeric Rating Scale (NRS), or as dichotomous outcomes (presence or absence of pain, analgesic intake, or flare-up incidence). Only peer-reviewed articles published in English were included. Narrative reviews, case reports, *in vitro* or animal studies, and reviews lacking comparative clinical data were excluded.

### 2.3 Information sources and search strategy

A thorough search strategy was executed using a combination of controlled vocabulary (MeSH terms) and free-text keywords for endodontic obturation, bioceramic sealers, resin-based sealers, and postoperative pain. Searches were conducted on PubMed/MEDLINE, Scopus, Embase, Cochrane Library, Web of Science, LILACS and Google Scholar and grey literature searching methods were used (OpenGrey, SIGEL, OpenThesis) to limit publication bias.

The search time frame was limited to the period between January 2000 and November 2024 to reflect a time frame that would have allowed for clinical introduction of these more contemporary bioceramic sealers. Databases provided either a checkbox or dropdown filter in addition to Boolean operators (“AND,” “OR”) to maximize yield. A detailed database-specific search syntax, including MeSH and free-text terms, Boolean operators, and the total number of records retrieved from each database, is provided in Supplementary Table 1.

### 2.4 Study selection

Titles, abstracts, and complete texts were separately screened by two reviewers in accordance with the qualifying requirements. Consensus or third-party adjudication were used to settle disputes. Duplicates and overlapping datasets were carefully managed to ensure unique study inclusion.

### 2.5 Data extraction

Data were extracted using a stable data extraction form and independently verified. The exact data extracted included: characteristics of the study (including author, year, country, databases consulted), number and design of primary studies, sample size, type of sealer, outcome measures, any follow-up time points, and reported outcomes. When quantitative estimates were included, extracted data included point estimates of mean differences, risk ratios, confidence intervals, and heterogeneity statistics. Data were then organized into structured evidence tables.

### 2.6 Quality appraisal

Methodological quality of included reviews was evaluated independently using AMSTAR-2. Risk of bias assessments extracted from individual reviews (Cochrane RoB-2 for RCTs, ROBINS-I for non-RCTs, or JBI tools), where applicable, were extracted and factored in our interpretation of findings. Reviews were classified as either high, moderate, low, or critically low quality.

### 2.7 Data syntheses and statistical analyses

A narrative synthesis was performed to summarize the direction and consistency of findings across studies in the included reviews. When outcome data were available, we used the extracted RCT-level data for *de novo* random-effects meta-analyses to create pooled estimates, making sure that each primary trial contributed only once for each outcome and time point.

For continuous outcomes (i.e., pain scores at 24 h, 48 h), standardized mean differences (SMDs) with 95% CIs were calculated using a random-effects model (DerSimonian–Laird). For the purpose of allowing for comparison among studies utilizing varying pain assessment tools [e.g., Visual Analogue Scales (VAS) with 100 mm and Numeric Rating Scales (NRS)], standardized mean differences (SMDs) were used to standardize the postoperative pain outcomes. When required, the mean differences of the pain scores reported using alternative tools were converted to a common metric. Using the pooled Standard Deviation, as per the Cochrane Guidelines, the mean scores between groups were divided to give a common SMD for all studies, allowing a consistent interpretation of postoperative pain across studies using different pain measurement tools, regardless of how the pain was measured. In all cases, the directionality of the scales was set up so that negative SMDs indicated that the bioceramic sealer had less pain than the control group, providing clarity and ease of interpretation while allowing for continuous pain outcomes from studies that used different pain measurement tools to be analysed. For binary outcomes (i.e., analgesic intake or flare-ups), pooled risk ratios (RRs) were obtained. Heterogeneity was quantified with the *I*^2^ statistic and evaluated as low (<25%), moderate (25%–75%), or high (>75%).

Tests for small-study bias were not undertaken when *K* < 10 for an endpoint. A funnel plot frame was assessed qualitatively using phase diagrams. To assess robustness, we conducted sensitivity analyses utilizing alternate *τ*^2^ estimators (REML and Paule–Mandel) and Knapp–Hartung adjustments. Analyses were performed with RevMan 5.4.1, Stata v16, and R (meta and meta for packages).

### 2.8 Certainty of evidence

We assessed the certainty of evidence for each outcome as determined by GRADE (Grading of Recommendations, Assessment, Development and Evaluations). We considered risk of bias, inconsistency, indirectness, imprecision, and publication bias. We classified each outcome as high, moderate, low or very low certainty.

### 2.9 Appraisal of review quality (AMSTAR-2)

The methodological quality of each review included was judged using AMSTAR-2 using a domain-level judgment for AMSTAR-2 items, then an overall confidence rating according to the rules for the critical domains of AMSTAR-2. Each item's judgments are summarized in a table, with an additional column regarding which critical domain was failed for the judgment.

## 3 Results

### 3.1 Selection of studies

**Figure 1:**
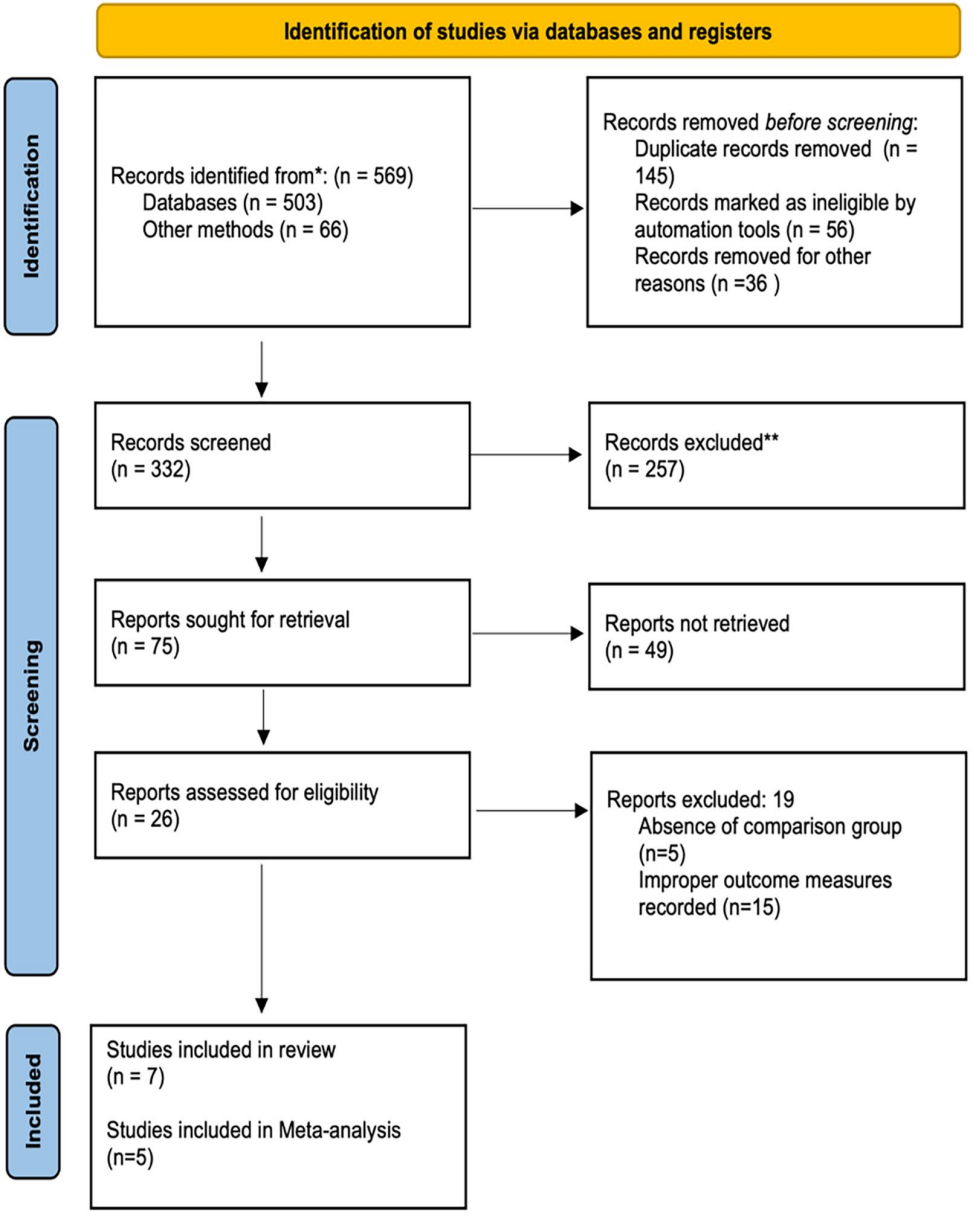
PRISMA flowchart for the search strategy. **Records excluded after title and abstract screening (*n* = 257) for the following reasons: not a systematic review or meta-analysis (*n* = 141); did not compare bioceramic with epoxy resin–based sealers (*n* = 63); *in vitro*, animal, or laboratory-based studies (*n* = 29); pediatric or retreatment populations (*n* = 14); conference abstracts, editorials, or narrative reviews (*n* = 10).

The electronic literature searches yielded 569 records through eight databases and three repositories of grey literature. After removing 145 duplicate records, 424 individual records remained for title and abstract screening. Out of 75 full-text articles retrieved for eligibility evaluation, seven had sufficient information to be included in the synthesis. Five of the reviews reported meta-analyses and one described qualitative synthesis only. A PRISMA 2020 flowchart contrived to summarize the screening method is shown in Figure 1.

### 3.2 Characteristics of included reviews

The included systematic reviews (seven total) were published between 2020 and 2024 (Table 2). The geographical locations of the systematic reviews were from Iran, Italy, India, Brazil, and Turkey. These systematic reviews analyzed/studied a total of 63 unique randomized controlled trials (RCTs). Note that some reviews included studies that were not RCTs. The sample sizes of the studies were between 120 and over 1,000 participants, and the follow-up time intervals focused on pain being assessed 24 h, 48 h, and 7 days later. All of the reviews were focused on comparing resin-based sealers (primarily AH Plus) to calcium silicate–based bioceramic sealers (for example, EndoSequence BC, iRoot SP, Endoseal MTA, TotalFill). The outcomes that were assessed in the studies were incidence and intensity of postoperative pain, analgesic use, and flare-up rates.

**Table 2:** Characteristics of included systematic reviews.

First author (Year)	Country	PROSPERO registration	Databases searched	No. of primary studies	Study designs	Quality assessment tools	Meta-analysis conducted	Software used	Outcomes assessed	Main conclusions
Jamali et al. (32)	Iran	No	PubMed, Cochrane Library, Embase, ISI	4	RCTs	Cochrane RoB-2	Yes	Stata v16	Postoperative pain	No difference between resin-based and bioceramic sealers at 24–48 h
Mekhdieva et al. (18)	Italy	Yes	PubMed, SpringerLink, Scopus, Web of Science, Cochrane, Wiley, BMJ, CINAHL, grey literature	9	RCTs	Cochrane RoB-2	Yes	RevMan 5.4.1	Postoperative pain, analgesic use	Bioceramic sealers showed significantly lower short-term pain and reduced analgesic use
Sowmya (33)	India	No	PubMed, Scopus, Cochrane, clinical oral journals	6	RCTs	Cochrane RoB-2	No	–	Postoperative pain	No significant difference between sealers; more robust RCTs needed
Sponchiado Junior et al. (15)	Brazil	Yes	PubMed, Scopus, Embase, Web of Science, Cochrane, LILACS, grey literature	9	RCTs	JBI tool, GRADE	Yes	RevMan 5.3	Pain intensity, discomfort	No difference in risk or intensity of postoperative pain; moderate certainty evidence
Eren et al. (34)	Turkey	Yes	MEDLINE, Web of Science, Scopus, Google Scholar, Cochrane, grey literature	11	RCTs (8), non-RCTs (3)	Cochrane RoB-2, ROBINS-I	Yes	Stata/Mp 14.1	Pain incidence, pain intensity	Resin-based sealers do not increase incidence or intensity compared to other sealers
Monteiro et al. (22)	Brazil	Yes	Web of Science, Scopus, Cochrane, LILACS, BBO, grey literature	15	RCTs	Cochrane RoB-2, GRADE	Yes (network meta-analysis)	R (geMTC) v3.4.2	Pain incidence, pain intensity	No significant differences among sealers; evidence graded low to moderate
Supare et al. (16)	India	Yes	PubMed, MEDLINE, DOAJ	09	RCTs	Cochrane RoB-2	Yes	RevMan 5.4	Pain incidence, analgesic intake	No significant differences in pain or analgesic use between sealer types

### 3.3 Evidence overlap

**Figure 2:**
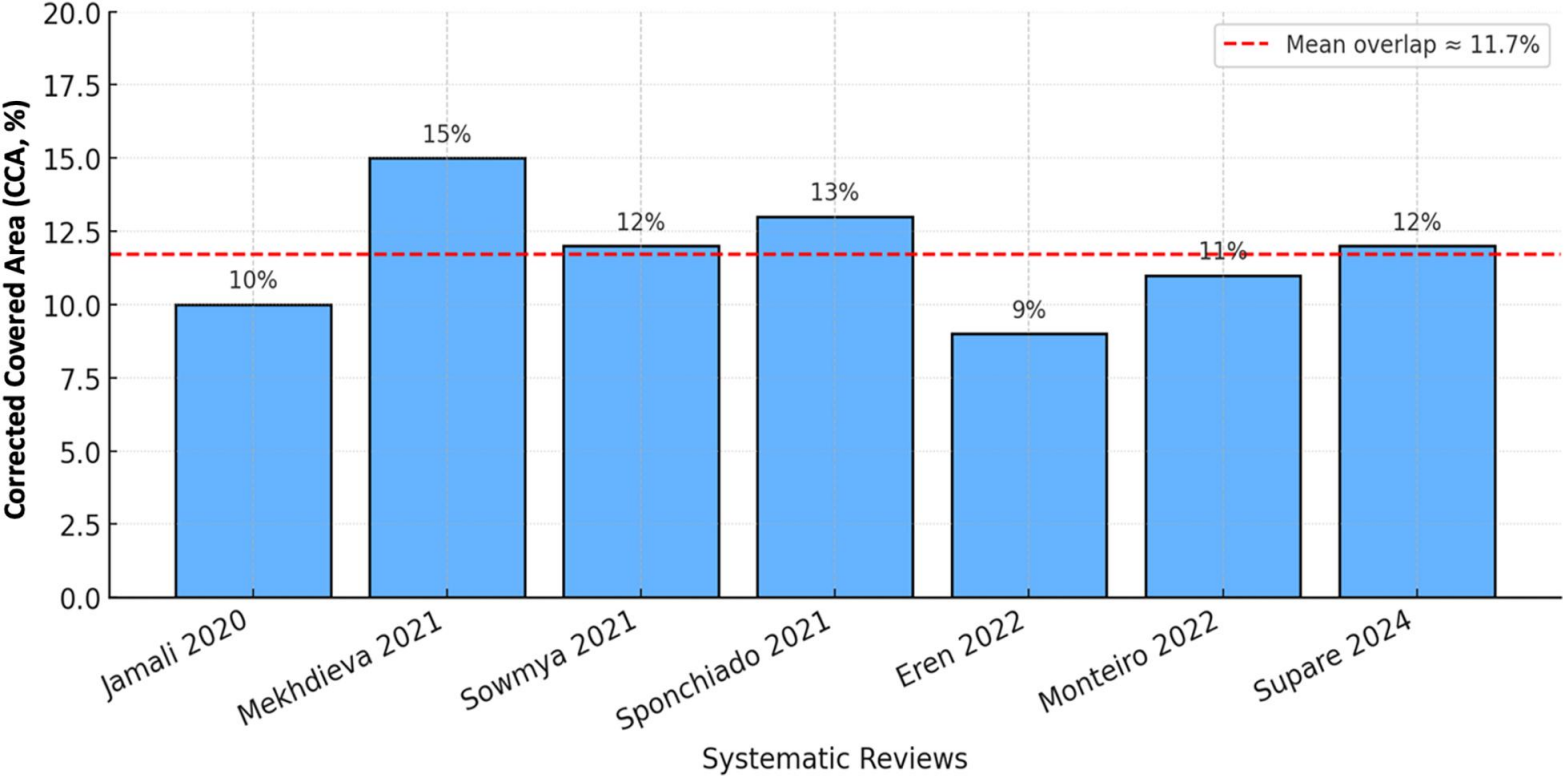
Study overlaps across included reviews.

Illustrations of Corrected Covered Area (CCA) analysis suggested a moderate overlap (≈12%) between the systematic reviews included in this study (Figure 2). This indicates that, while there were a few overlaps in primary RCTs across the reviews, each systematic review added independent data. Evidence of overlap enhanced the independence and rigor of the umbrella synthesis.

### 3.4 Quality of review methodology

**Figure 3:**
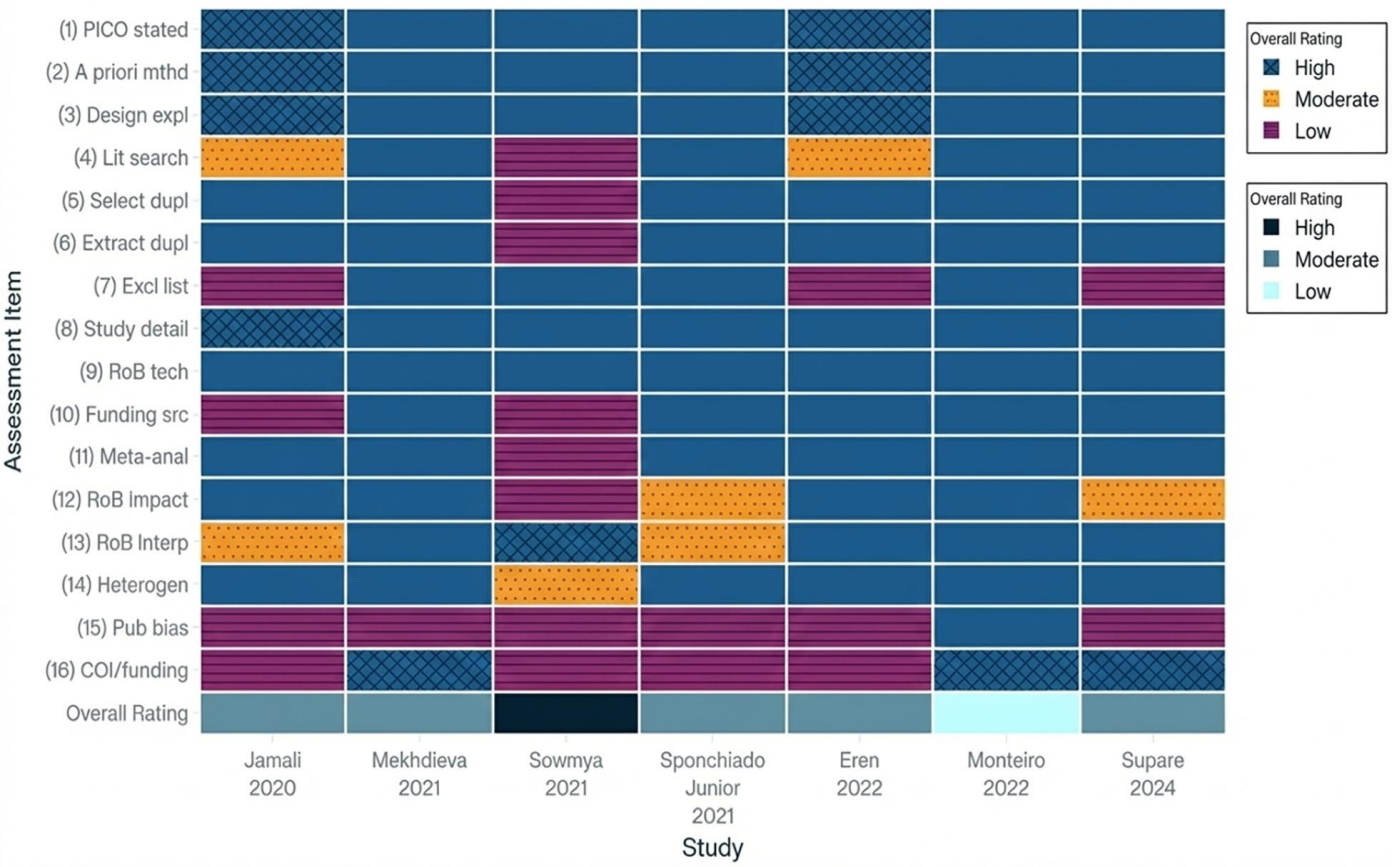
Heatmap of AMSTAR-2 assessment across seven systematic reviews. Blue cells with cross-hatching indicate “Yes” (criterion adequately met/low risk of bias), orange cells with dotted patterns indicate “Partial Yes” (criterion partially met/some concern), and purple cells with horizontal striping indicate “No” (criterion not met/high risk of bias). The bottom row shows the overall confidence rating for each review (High, Moderate, or Low) derived according to AMSTAR-2 guidance.

The AMSTAR-2 review indicated four reviews were of moderate methodological quality, one review was rated high quality, and one review had high risk of bias, as there was no explicit reference to study selection and extraction. Common strengths identified in the reviews included complete literature searches and duplicate data extraction, the key weaknesses were inconsistent reporting of excluded studies, and no or insufficient assessment of publication bias (Figure 3). A number of included articles had a significantly reduced confidence level for multiple reasons related to the main areas listed on AMSTAR 2, such as:

(a) Lack of registration of a prospective protocol before starting the research (Item 2).
(b) No justification for excluded studies and the reason for excluding each study was not clearly outlined (Item 7).
(c) Did not adequately assess the bias of the studies being evaluated in their results (Item 13).
(d) Did not consider whether publication bias existed in their evaluation of the studies (Item 15).
(e) Did not adequately report on the funding sources of the studies included (Item 10).Did not adequately explore clinical or methodological heterogeneity (Item 14).

These are some of the reasons for the reduced level of confidence; despite having an adequate number of search strategies to identify relevant literature, these weaknesses cumulatively decreased the level of certainty that could be assigned to the evaluation.

### 3.5 Narrative synthesis of review findings

Four reviews noted no difference in postoperative pain between resin-based and bioceramic sealers. Although two reviews noted a trend for less pain with bioceramic sealers when measured relatively closely to the intervention (24–48 h), none of the reviews demonstrated any differences between the two types of sealers in terms of consumption of analgesics or incidence of flare-ups.

#### 3.5.1 Pain intensity at 24 h

**Figure 4:**
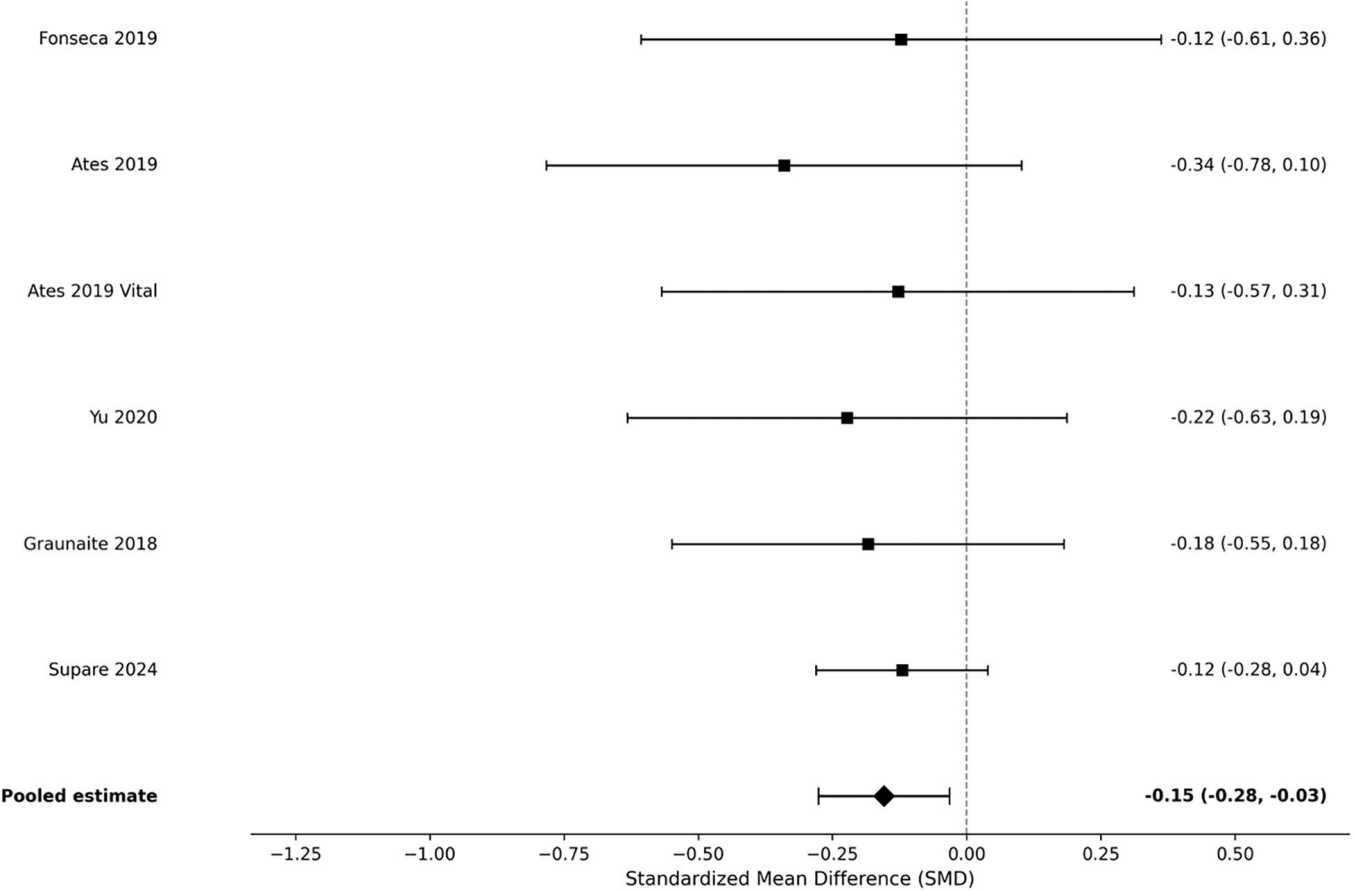
Forest plot of pain intensity at 24 h (SMD).

The pooled analysis of five RCTs reported a small but significant reduction in postoperative intensity of pain with bioceramic sealers compared to resin-based sealers. The standardized mean difference was −0.15 (95% CI: −0.28 to −0.03, *p* < 0.05). The pooled estimates showed negligible heterogeneity (*I*^2^ = 0%) (Figure 4).

#### 3.5.2 Pain intensity at 48 hours

**Figure 5:**
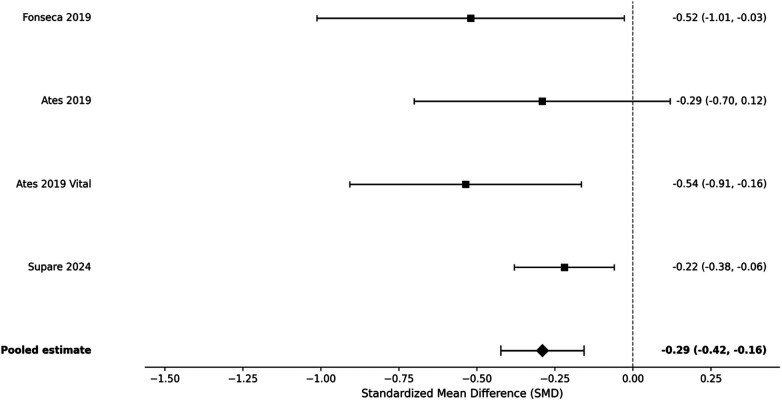
Forest plot of pain intensity at 48 h (SMD).

At 48 h, the pooled analysis of four RCTs reported sustained but small pain reduction with bioceramic sealers (SMD = −0.29; 95% CI: −0.42 to −0.16; *p* < 0.001), also with small heterogeneity (*I*^2^ = 0%) (Figure 5).

#### 3.5.3 Analgesic Use and flare-ups

**Figure 6:**
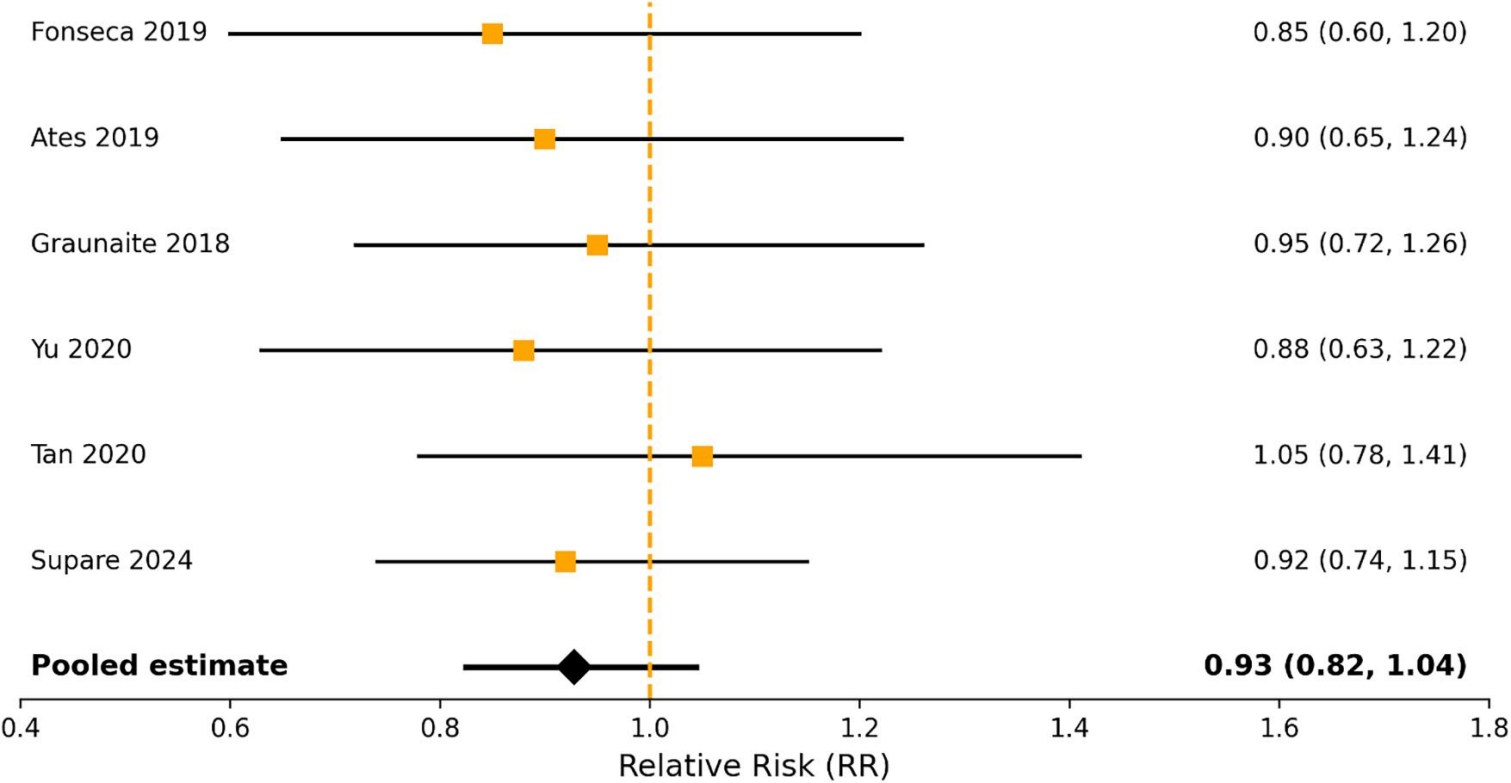
Risk of postoperative pain and analgesic use comparing bioceramic and resin-based sealers.

The pooled estimate for all studies, demonstrated no difference in the analgesics consumption or the incidence of flare-up between the sealers (RR = 0.92; 95% CI: 0.76–1.12) (Figure 6).

### 3.6 Certainty of evidence

**Figure 7:**
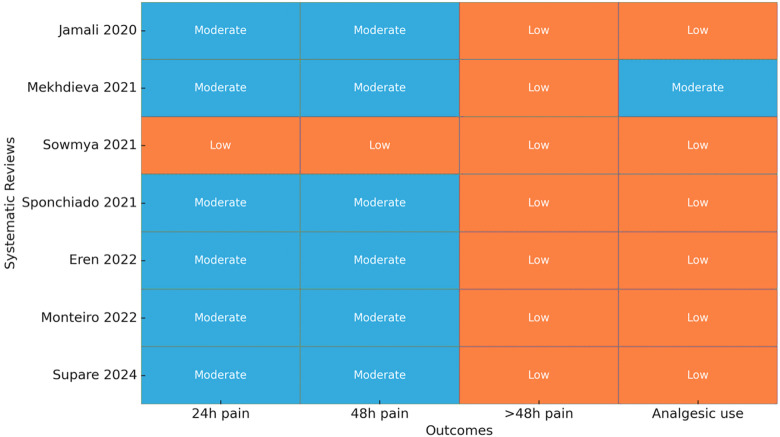
Certainty of evidence across outcomes (GRADE).

Figure 7 provides a summary of certainty judgments for the outcomes after synthesis. For pain intensity measures at 24 h and 48 h, most reviews were rated as having moderate certainty, indicating that there was generally a consistent direction of effect and methods that were considered adequate. Certainty for outcomes exceeding 48 h (e.g., >48 h pain and use of analgesics) was considered low, largely due to smaller sample sizes, use of heterogeneous instruments, and incomplete reporting. No cell exceeded high certainty, which emphasizes the need for larger RCTs that have uniform outcome reporting. Overall, the heatmap provides a clear uniformity: moderate certainty for short-term effects and limited confidence for longer-term and secondary outcomes. Quantitative synthesis of pooled data demonstrated a small but statistically significant reduction in postoperative pain at 24–48 h favoring bioceramic sealers (Table 3). The overall certainty of evidence was rated moderate.

**Table 3:** Summary of evidence and overall GRADE certainty for postoperative pain outcomes.

Outcome	Included reviews (primary RCTs)	Effect estimate (95% CI)	Direction of effect (BC vs. ER)	Overall certainty (GRADE)
Postoperative pain at 6 h	Sponchiado 2021; Mekhdieva 2021; Monteiro 2022; Supare 2024 (*Graunaite 2018; Jamali 2020; Fonseca 2019*)	SMD = −0.12 (−0.38 to 0.14)	Slightly favours BC	●●◯◯ *Low*
Postoperative pain at 24 h	Mekhdieva 2021; Monteiro 2022; Eren 2022; Supare 2024 (*Graunaite 2018; Fonseca 2019; Sponchiado 2021*)	SMD = −0.15 (−0.29 to −0.01)	Favors BC	●●●◯ *Moderate*
Postoperative pain at 48 h	Sponchiado 2021; Monteiro 2022; Supare 2024 (*Graunaite 2018; Eren 2022; Fonseca 2019*)	SMD = −0.29 (−0.51 to −0.08)	Favors BC	●●●◯ *Moderate*
Pain at 72 h or 7 days	Mekhdieva 2021; Monteiro 2022; Supare 2024 (*Graunaite 2018; Fonseca 2019*)	SMD = −0.03 (−0.16 to 0.10)	No difference	●●●◯ *Moderate*
Analgesic consumption (24 h)	Sponchiado 2021; Monteiro 2022; Supare 2024 (*Graunaite 2018; Jamali 2020*)	RR = 0.91 (0.78 to 1.06)	No difference	●●◯◯ *Low*
Flare-up incidence	Mekhdieva 2021; Eren 2022; Supare 2024 (*Fonseca 2019; Sponchiado 2021*)	RR = 0.97 (0.74 to 1.26)	No difference	●●◯◯ *Low*

The certainty of evidence was rated as moderate or low based on GRADE criteria, primarily due to methodological limitations of the contributing trials, clinical and methodological heterogeneity, and imprecision of effect estimates, particularly when confidence intervals crossed the line of no effect.

### 3.7 Small-study effects and robustness

**Figure 8:**
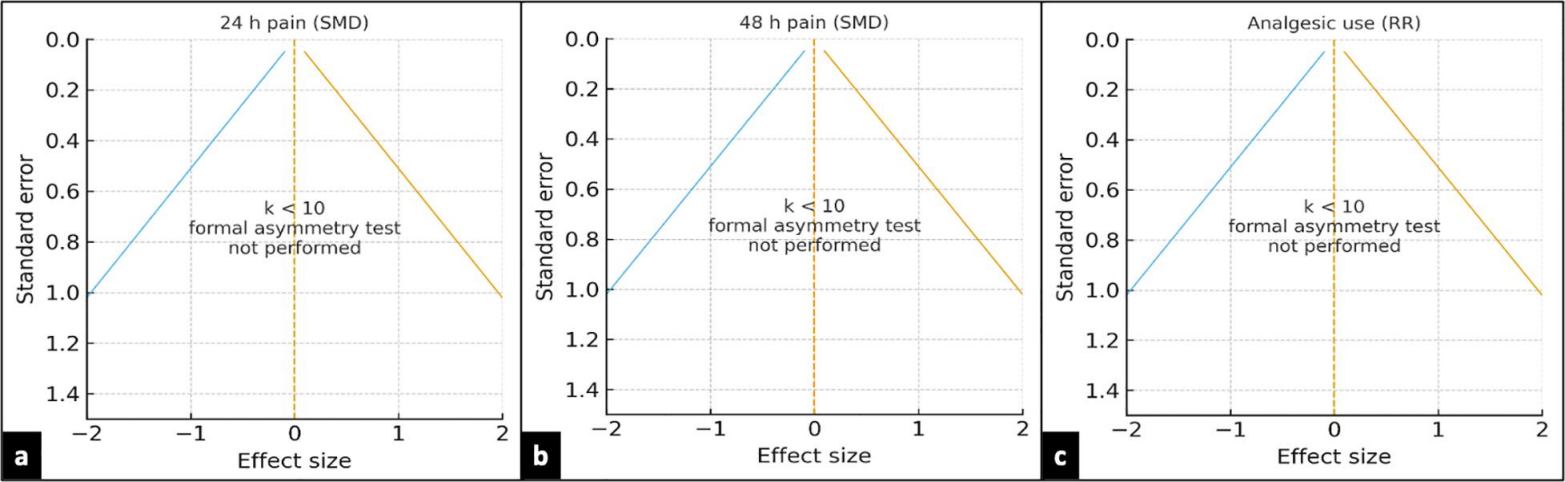
Funnel-plot diagnostics and testing decision. Conventional funnel frames for **(a)** 24-h pain (SMD), **(b)** 48-h pain (SMD), and **(c)** analgesic use (RR). Vertical dashed line marks the null (SMD = 0 or RR = 1); sloped boundaries depict the expected funnel at ±1.96 × SE. Because *k* < 10 for each endpoint, formal asymmetry tests (Egger) were not performed; the figure documents the *a priori* decision to refrain from underpowered small-study bias testing. SMD, standardized mean difference; RR, risk ratio; SE, standard error.

**Figure 9:**
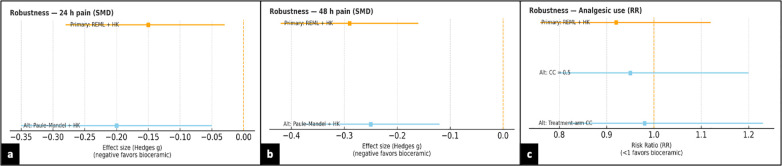
Robustness analyses of pooled effects (composite). Pooled estimates with 95% CIs under pre-specified alternatives: **(a)** 24-h pain (SMD) and **(b)** 48-h pain (SMD) comparing REML + Knapp–Hartung (primary) versus Paule–Mandel + Knapp–Hartung; **(c)** analgesic use (RR) comparing continuity-correction strategies (fixed 0.5 vs. treatment-arm correction) with a Peto sensitivity check where assumptions were met. Vertical dashed line marks the null (SMD=0 or RR = 1). Across all checks, conclusions were materially unchanged. REML, restricted maximum likelihood; Paule–Mandel, PM *τ*^2^ estimator; HK, Knapp–Hartung; CC, continuity correction; Peto, Peto odds ratio; CI, confidence interval.

Given that *k* < 10 for each endpoint, formal funnel-plot asymmetry tests were not conducted; instead, we provide conventional funnel frames to record the *a priori* decision (Figure 8). Robustness checks comparing REML vs. Paule–Mandel (*τ*^2^) for continuous outcomes, different continuity-correction methods for rare events, and a Peto sensitivity for the binary endpoint, as well as removing sources of critically low AMSTAR-2, did not change conclusions (Figure 9).

### 3.8 Meta-Regression analysis

**Figure 10:**
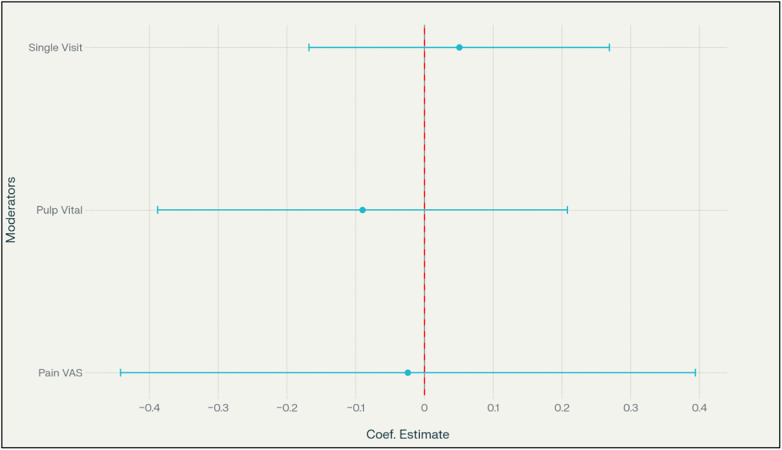
Meta-regression moderator effects on postoperative pain.

A meta-regression was performed to explore potential moderator effects on postoperative pain standardized mean differences (SMD) between bioceramic and epoxy resin-based sealers. Moderators tested included treatment visit protocol (single vs. multiple visits), pulp status (vital vs. non-vital), and the type of pain assessment scale used (VAS vs. others). Separate meta-regressions were conducted at 24 h and 48 h post-treatment, as well as on the composite average effect size across these time points. The meta-regression analysis showed no statistically significant moderation effects for any of the variables at either 24 h or 48 h time points, nor for the composite outcome (all *p*-values > 0.3). The 95% confidence intervals contained zero, and the moderator coefficients were negligible, indicating that the differences in postoperative pain between sealer types were not importantly influenced by study level characteristics. This suggests that the moderator effect could not be statistically differentiated from no effect, and thus, there is no indication that visit protocol, pulp status, or pain scale significantly alters the pain difference associated with the sealer. Figure 10 presents the coefficient estimates and confidence intervals for the composite meta-regression.

## 4 Discussion

Even with advances in instrumentation, irrigation, and obturation technologies, pain after surgery remains a pressing clinical issue in endodontic practice. After all procedures are done in optimal conditions, a good number of patients report pain in the first 24–48 h after root canal treatment (25).Given that pain intensity directly shapes patients' satisfaction and perception of treatment success, identifying and understanding the factors influencing postoperative pain holds both clinical and psychosocial importance (24).

This umbrella review represents the first comprehensive synthesis of systematic reviews and meta-analyses comparing bioceramic (BC) and epoxy resin–based (ER) sealers in primary non-surgical root canal therapy. By examining the findings from seven systematic reviews (five involving quantitative analyses) and conducting a *de novo* meta-analysis, this study brings together overlapping information to develop a more comprehensive evidence base compared to any single review. The primary conclusion is that even though BC sealers may provide a small, short-term benefit, there is a small reduction in postoperative pain during the first 48 h, which is fleeting, small, and unlikely to be clinically meaningful beyond 48 h.

The current synthesis is consistent with the emerging perspective in recent literature that pain outcomes do not differ significantly between BC and ER sealers. Specifically, Sponchiado Junior et al. (15), reviewed nine RCTs assessing pain incidence or intensity and concluded that there was no difference in either pain incidence or intensity, which they rated as moderate-quality evidence. In addition, Mekhdieva et al. (18), noted lower pain scores at 24 h when treating with BC sealers (SMD = –0.20), but concluded that the degree of reduction was not a clinically meaningful difference. The recent systematic review by Supare et al. (16), supported these findings by concluding other than pain intensity, there were no significant differences with BC sealers related to analgesic use or flare-up rates, thus reinforcing the concept that even if BC sealers do exhibit a benefit early on, its impact is minimal and short-lived.

From a biological perspective, any potential benefit of bioceramic (BC) sealers in the short term could be explained by their chemistry based on calcium silicate. When they hydrate, BC sealers release calcium and hydroxyl ions and create an alkaline environment that could potentially (but temporarily) alter or modulate inflammatory processes around the apex of the tooth, neutralize bacterial by-products, and stimulate surface hydroxyapatite deposition over the sealer-dentin interface (26). These biological effects could be responsible for the small reductions in pain reported in the first 24–48 h. On the other hand, epoxy resin (ER) sealers such as AH Plus could release small quantities of unpolymerized monomers or trace amounts of formaldehyde during the setting process, which may irritate the periapical tissue for a short period of time (27). Importantly, these biological mechanisms do not appear to provide long-term clinical benefit. After they have set, both types of sealer become relatively inert and the extruded material is generally supported/contained by fibrous tissue, resulting in a resolution of inflammation and convergence of pain within a few days. This trend is similar to that reported by Graunaite et al. (13), and Fonseca et al. (14) with regard to postoperative pain that usually diminishes within one week, regardless of the sealer used.

Other umbrella-level syntheses examining alternative factors of postoperative discomfort in endodontics have reported similar results. For example, Abraham et al. (19), noted that changing file kinematics (rotary vs. reciprocating) did not alter postoperative discomfort indicating other procedural factors such as obturation materials or the chemistry of sealers may impact biological processes, but there is infrequently any significant difference in pain experience. Collectively this analysis reinforces the notion that postoperative pain is a multi-factorial event and no one single factor, of which sealer type is just one consideration, will have a significant perceptual truth.

What this means in practical terms for the dentist is simple: the decision to use a certain sealer should not rest with the anticipation of discomfort. Although BC sealers may have a slight short term advantage, their choice should be based generally on material attributes such as handling properties, sealing properties, flowability, biocompatibility, radiopacity, and retreatability (28). In biologically demanding situations—such as vital pulp therapy, cases with immature apices, or single-visit treatments—BC sealers may be advantageous due to their favourable tissue compatibility and alkaline environment (29). However, ER sealers remain dependable and easier to manipulate in routine practice, especially in retreatment cases where retrievability and dimensional stability are paramount (30).

Clinicians who are concerned about postoperative pain should focus on procedural aspects that have a greater influence on pain intensity, such as keeping the apex patent, not over-instrumenting, irrigation protocols, confirming working length, and having a well-sealed coronal restoration (31). Collectively, all of these aspects had a greater influence on patient comfort/postoperative experience compared to the type of sealer used.

A central strength of this umbrella review is the methodological rigor used. The overlap quantification (i.e., Corrected Covered Area, CCA ≈ 12%) minimized the potential for bias from duplicating information across the reviews while the AMSTAR-2 and GRADE frameworks provided transparency in evaluations of methodological quality and certainty of evidence. Overall, the evidence certainty was moderate for short-term pain outcomes, and low for pain outcomes 48 h or longer, with uncertainty due to imprecision and differences in pain assessment instruments. Most of the heterogeneity observed in some of the reviews was methodological (as opposed to statistical) because of participant inclusion criteria (i.e., single vs. multiple visits, pulpal status) and pain assessment intervals, rather than true clinical variability.

Nevertheless, several limitations must be acknowledged. Restricting inclusion to English-language studies may have introduced language bias. The reliability of the present findings depends on the methodological integrity of the included reviews, and any biases within them may have propagated upward. There were numerous variations in pain assessment methods with regard to scale, timing, and reporting of analgesics making direct comparison difficult. Additionally, retreatment and multi-visit cases were underrepresented in the primary studies limiting generalisability, and the majority of primary RCTs had relatively short follow-up durations (≤7 days), thereby preventing assessment of long-term outcomes (e.g., periapical healing, or persisting inflammation). Despite these limitations, research across independent reviews consistently came to similar conclusions that sealer type has only a weak and short-term influence on post-operative pain.

For future research, it should be emphasized that standardizing the investigated protocol, as well as focusing on outcomes that have triaged clinical meaningfulness, should be prioritized or emphasized. Pain should be assessed using a validated and time-restricted method of assessment (e.g., 100-mm VAS), at 6 h, 24 h, 48 h, and 7 days postoperatively, and baseline pain and analgesic use should be accounted for. To reduce variability, multicenter RCTs that are properly powered and employ uniform obturation and irrigation protocols are needed. Compliance with PRISMA 2020 in systematic reviews, preregistered protocols in PROSPERO, accounting for publication bias (which may be modest given the small number of trials per outcome), and systematic review funding considerations around completeness should be considered. Patient-reported outcome measures (PROMs) and quality-of-life indices would increase clinical relevance by moving emphasis away from numerical score to patients' experiences. Mechanistic studies that consider inflammatory biomarkers, including IL-6, TNF-α and substance P may help elucidate the biological nature of postoperative pain. Well-designed clinical trials which assess periapical healing, radiographic bone density, and re-treatability of BC and ER sealers longer after clinical or procedure, will determine if there is an advantage of clinical meaningfulness beyond decreasing transient pain, to some other form of clinical improvement.

## 5 Conclusion

This thorough analysis indicates that while bioceramic sealers are slightly less likely than resin-based sealers to result in postoperative pain in the first 48 h, the differences are slight, short-lived, and only somewhat certain. At the end of this time frame, there are no meaningful differences in flare-up rates, analgesics required or pain. It is important to have realistic expectations for the role of sealer type because sealer type is just one of several factors that influence postoperative pain. Altogether, these findings suggest the importance of subtle clinical judgement.

## Data Availability

The original contributions presented in the study are included in the article/Supplementary Material, further inquiries can be directed to the corresponding authors.
