# Supplementary material for: Evidence synthesis of postoperative pain with bioceramic vs. epoxy resin sealers: umbrella review of randomized trials within existing systematic reviews
Source: Front Dent Med. 2026 Jan 16;6:1749298. doi: 10.3389/fdmed.2025.1749298 (PMC12880009; doi:10.3389/fdmed.2025.1749298)
Supplement: Supplementary file 1 [file Datasheet1.pdf]

National Institute for Health Research

University of York
Centre for Reviews and Dissemination

Systematic review

*Fields that have an **asterisk (*)** next to them means that they **must be answered. Word limits** are provided for each section. You will be unable to submit the form if the word limits are exceeded for any section. Registrant means the person filling out the form*.

This record cannot be edited because it has been marked as out of scope

1. * Review title.

Give the title of the review in English

Incidence of postoperative pain using bioceramic sealers and resin based sealers in root canal treatment among adults - An Umbrella review.

2. Original language title.

For reviews in languages other than English, give the title in the original language. This will be displayed with the English language title.

Incidence of postoperative pain using bioceramic sealers and resin based sealers in root canal treatment among adults - An Umbrella review

3. * Anticipated or actual start date.

Give the date the systematic review started or is expected to start.

01/11/2023

4. * Anticipated completion date.

Give the date by which the review is expected to be completed.

30/04/2024

5. * Stage of review at time of this submission.

This field uses answers to initial screening questions. It cannot be edited until after registration.

Tick the boxes to show which review tasks have been started and which have been completed.

Update this field each time any amendments are made to a published record.

The review has not yet started: Yes

Review stage	Started	Completed
Preliminary searches	No	No
Piloting of the study selection process	No	No
Formal screening of search results against eligibility criteria	No	No
Data extraction	No	No
Risk of bias (quality) assessment	No	No
Data analysis	No	No

Provide any other relevant information about the stage of the review here.

6. * Named contact.

The named contact is the guarantor for the accuracy of the information in the register record. This may be any member of the review team.

MRUNALI DAHIKAR

Email salutation (e.g. "Dr Smith" or "Joanne") for correspondence:

Dr DAHIKAR

7. * Named contact email.

Give the electronic email address of the named contact.

mrunalidahikar1997@gmail.com

8. Named contact address

Give the full institutional/organisational postal address for the named contact.

NAIR HOSPITAL DENTAL COLLEGE, 55, DR. ANANDRAO NAIR MARG, IN FRONT OF MARATHA MANDIR, MUMBAI CENTRAL, MUMBAI, 400008 , MAHARASHTRA, INDIA

9. Named contact phone number.

Give the telephone number for the named contact, including international dialling code.

9766912999

10. * Organisational affiliation of the review.

Full title of the organisational affiliations for this review and website address if available. This field may be completed as 'None' if the review is not affiliated to any organisation.

NAIR HOSPITAL DENTAL COLLEGE, MUMBAI

Organisation web address:

11. * Review team members and their organisational affiliations.

Give the personal details and the organisational affiliations of each member of the review team. Affiliation refers to groups or organisations to which review team members belong. **NOTE: email and country now MUST be entered for each person, unless you are amending a published record.**

Dr MRUNALI DAHIKAR. NAIR HOSPITAL DENTAL COLLEGE, MUMBAI
Dr ASHISH MANDWE. NAIR HOSPITAL DENTAL COLLEGE, MUMBAI
Dr KULVINDER SINGH BANGA. NAIR HOSPITAL DENTAL COLLEGE, MUMBAI
Dr AJINKYA PAWAR. NAIR HOSPITAL DENTAL COLLEGE, MUMBAI

12. * Funding sources/sponsors.

Details of the individuals, organizations, groups, companies or other legal entities who have funded or sponsored the review.

NO

Grant number(s)

State the funder, grant or award number and the date of award

13. * Conflicts of interest.

List actual or perceived conflicts of interest (financial or academic).

None

14. Collaborators.

Give the name and affiliation of any individuals or organisations who are working on the review but who are not listed as review team members. **NOTE: email and country must be completed for each person, unless you are amending a published record.**

15. * Review question.

State the review question(s) clearly and precisely. It may be appropriate to break very broad questions down into a series of related more specific questions. Questions may be framed or refined using PI(E)COS or similar where relevant.

Is there any difference in incidence of postoperative pain using bioceramic sealers and resin based sealers in root canal treatment among adults - An Umbrella review

16. * Searches.

State the sources that will be searched (e.g. Medline). Give the search dates, and any restrictions (e.g. language or publication date). Do NOT enter the full search strategy (it may be provided as a link or attachment below.)

A systematic search following the principle of systematic review will be carried out in
the Cochrane Central Register of Controlled Trials (CENTRAL), MEDLINE, Google

Scholar, PubMed using MeSH words, text words and Boolean operators. The articles in the English language will be considered. The period of publication considered will be up to 30-09-2023.

17. URL to search strategy.

Upload a file with your search strategy, or an example of a search strategy for a specific database, (including the keywords) in pdf or word format. In doing so you are consenting to the file being made publicly accessible. Or provide a URL or link to the strategy. Do NOT provide links to your search **results**.

Alternatively, upload your search strategy to CRD in pdf format. Please note that by doing so you are consenting to the file being made publicly accessible.

Do not make this file publicly available until the review is complete

18. * Condition or domain being studied.

Give a short description of the disease, condition or healthcare domain being studied in your systematic review.

Post-operative pain is pain of any degree that occurs after initiation of root canal treatment. The causes of postoperative pain can be classified as mechanical, chemical and/or microbiological injuries to the peri-radicular tissues.

19. * Participants/population.

Specify the participants or populations being studied in the review. The preferred format includes details of both inclusion and exclusion criteria.

Adult healthy patients undergoing non-surgical root canal treatment
[illegible] sex, type of endodontic diagnosis, or type of tooth treated without restrictions

20. * Intervention(s), exposure(s).

Give full and clear descriptions or definitions of the interventions or the exposures to be reviewed. The preferred format includes details of both inclusion and exclusion criteria.

Root canal treatment done using bioceramics based sealers

21. * Comparator(s)/control.

Where relevant, give details of the alternatives against which the intervention/exposure will be compared (e.g. another intervention or a non-exposed control group). The preferred format includes details of both inclusion and exclusion criteria.

Root canal treatment done using resin based sealers

22. * Types of study to be included.

Give details of the study designs (e.g. RCT) that are eligible for inclusion in the review. The preferred format includes both inclusion and exclusion criteria. If there are no restrictions on the types of study, this should be stated.

Inclusion

Systematic review and meta-analysis of randomized controlled trials, non-RCTs, cross-

sectional studies.

Exclusion:

Clinical trials, prospective studies, cross-sectional studies, Review reports, case series, in-

vitro and animal studies will be excluded

23. Context.

Give summary details of the setting or other relevant characteristics, which help define the inclusion or exclusion criteria.

Inclusion Criteria

• Studies published till 30/09/2023

• Studies published in English language only.

• Studies with full-text articles only will be included.

Exclusion Criteria

• Studies not fully available in the database.

• In-vitro and animal studies

• Studies not mentioning sample size

• Studies including population with endodontic or periapical lesion

24. * Main outcome(s).

Give the pre-specified main (most important) outcomes of the review, including details of how the outcome is defined and measured and when these measurement are made, if these are part of the review inclusion criteria.

Post-operative pain, analgesic use

Measures of effect

Please specify the effect measure(s) for you main outcome(s) e.g. relative risks, odds ratios, risk difference, and/or 'number needed to treat.

Mean, standard deviation, percentages

25. * Additional outcome(s).

List the pre-specified additional outcomes of the review, with a similar level of detail to that required for main outcomes. Where there are no additional outcomes please state 'None' or 'Not applicable' as appropriate to the review

NONE

Measures of effect

Please specify the effect measure(s) for you additional outcome(s) e.g. relative risks, odds ratios, risk difference, and/or 'number needed to treat.

26. * Data extraction (selection and coding).

Describe how studies will be selected for inclusion. State what data will be extracted or obtained. State how this will be done and recorded.

Titles and/or abstracts of studies retrieved using the search strategy and those from additional
sources will be screened independently by two review authors to identify studies that

potentially meet the inclusion criteria outlined above. The full texts of these potentially

eligible studies will be retrieved and independently assessed for eligibility by two review

team members. For the assessment of each publication, spreadsheets will be used. Data will

be extracted using a standardized form. Both the authors will compare each other and confirm

the data.

Two review authors will extract data independently and third author will cross check the data.

Discrepancies will be identified and resolved through discussion (with a third author where necessary). The data to be extracted will include: author's name, year of publication, study design, sample size, age group, Intervention description, comparison description if any, outcome measures and confounding factors if any.

27. * Risk of bias (quality) assessment.

State which characteristics of the studies will be assessed and/or any formal risk of bias/quality assessment tools that will be used.

Quality assessment will be done using AMSTAR-2 tool consisting of 11 questions under 7
Critical domains. The overall confidence in the results of the review is classified as high,

moderate, low and critically low

28. * Strategy for data synthesis.

Describe the methods you plan to use to synthesise data. This **must not be generic text** but should be **specific to your review** and describe how the proposed approach will be applied to your data. If meta-analysis is planned, describe the models to be used, methods to explore statistical heterogeneity, and software package to be used.

If similar kind of sufficient studies will be identified then Meta-analysis will be carried out.
The RevMan software will be used for the analysis. If there are clinical and methodological

differences in the outcome of different studies, the random effects or fixed effect model will be used depending on the heterogeneity of the included studies and meta-analysis will be planned. The publication bias will be tested by funnel plot. The statistical heterogeneity will be examined using the I^2 statistic. In the presence of substantial heterogeneity, the reasons for the same will be explored and sensitivity analysis will be conducted

29. * Analysis of subgroups or subsets.

State any planned investigation of ‘subgroups’. Be clear and specific about which type of study or participant will be included in each group or covariate investigated. State the planned analytic approach.

Analysis of sub-groups is not planned yet.

30. * Type and method of review.

Select the type of review, review method and health area from the lists below.

Type of review

Cost effectiveness

No

Diagnostic

No

Epidemiologic

No

Individual patient data (IPD) meta-analysis

No

Intervention

No

Living systematic review

No

Meta-analysis

Yes

Methodology

No

Narrative synthesis

No

Network meta-analysis

No

Pre-clinical

No

Prevention

No

Prognostic

No

Prospective meta-analysis (PMA)

No

Review of reviews

No

Service delivery

No

Synthesis of qualitative studies

No

Systematic review
Yes

Other
No

Health area of the review

Alcohol/substance misuse/abuse
No

Blood and immune system
No

Cancer
No

Cardiovascular
No

Care of the elderly
No

Child health
No

Complementary therapies
No

COVID-19
No

Crime and justice
No

Dental
Yes

Digestive system
No

Ear, nose and throat
No

Education
No

Endocrine and metabolic disorders
No

Eye disorders

No

General interest

No

Genetics

No

Health inequalities/health equity

No

Infections and infestations

No

International development

No

Mental health and behavioural conditions

No

Musculoskeletal

No

Neurological

No

Nursing

No

Obstetrics and gynaecology

No

Oral health

No

Palliative care

No

Perioperative care

No

Physiotherapy

No

Pregnancy and childbirth

No

Public health (including social determinants of health)

No

Rehabilitation

No

Respiratory disorders

No

Service delivery

No

Skin disorders

No

Social care

No

Surgery

No

Tropical Medicine

No

Urological

No

Wounds, injuries and accidents

No

Violence and abuse

No

31. Language.

Select each language individually to add it to the list below, use the bin icon to remove any added in error.
English

There is not an English language summary

32. * Country.

Select the country in which the review is being carried out. For multi-national collaborations select all the countries involved.

India

33. Other registration details.

Name any other organisation where the systematic review title or protocol is registered (e.g. Campbell, or The Joanna Briggs Institute) together with any unique identification number assigned by them. If extracted data will be stored and made available through a repository such as the Systematic Review Data Repository (SRDR), details and a link should be included here. If none, leave blank.

34. Reference and/or URL for published protocol.

If the protocol for this review is published provide details (authors, title and journal details, preferably in Vancouver format)

Add web link to the published protocol.

Or, upload your published protocol here in pdf format. Note that the upload will be publicly accessible.

No I do not make this file publicly available until the review is complete

Please note that the information required in the PROSPERO registration form must be completed in full even if access to a protocol is given.

35. Dissemination plans.

Do you intend to publish the review on completion?

No

Give brief details of plans for communicating review findings.?

36. Keywords.

Give words or phrases that best describe the review. Separate keywords with a semicolon or new line. Keywords help PROSPERO users find your review (keywords do not appear in the public record but are included in searches). Be as specific and precise as possible. Avoid acronyms and abbreviations unless these are in wide use.

37. Details of any existing review of the same topic by the same authors.

If you are registering an update of an existing review give details of the earlier versions and include a full bibliographic reference, if available.

38. * Current review status.

Update review status when the review is completed and when it is published.New registrations must be ongoing so this field is not editable for initial submission.

Please provide anticipated publication date

Review_Ongoing

39. Any additional information.

Provide any other information relevant to the registration of this review.

40. Details of final report/publication(s) or preprints if available.

Leave empty until publication details are available OR you have a link to a preprint (NOTE: this field is not editable for initial submission). List authors, title and journal details preferably in Vancouver format.

Give the link to the published review or preprint.
