# Supplementary material for: Evidence synthesis of postoperative pain with bioceramic vs. epoxy resin sealers: umbrella review of randomized trials within existing systematic reviews
Source: Front Dent Med. 2026 Jan 16;6:1749298. doi: 10.3389/fdmed.2025.1749298 (PMC12880009; doi:10.3389/fdmed.2025.1749298)
Supplement: Supplementary file 2 [file Table1.docx]

| **Database** | **Search strategy** | **Records retrieved** |
| --- | --- | --- |
| **PubMed/MEDLINE** | (((("dental pulp cavity"[MeSH Terms] OR ("root canal"[All Fields]) OR ("endodontic treatment"[All Fields])) AND ("therapy"[MeSH Terms] OR "treatment"[All Fields])) AND (bioceramic[All Fields] AND sealers[All Fields])) AND (resin[All Fields] AND sealers[All Fields])) AND ("pain, postoperative"[MeSH Terms] OR "postoperative pain"[All Fields] OR "pain intensity"[All Fields])) AND ("systematic review"[All Fields] OR "meta-analysis"[All Fields]) | 145 |
| **Embase** | ('root canal therapy'/exp OR 'endodontic treatment') AND ('bioceramic sealer' OR 'calcium silicate sealer') AND ('resin-based sealer' OR 'epoxy resin sealer') AND ('postoperative pain' OR 'pain incidence' OR 'pain intensity') AND ('systematic review' OR 'meta-analysis') | 102 |
| **Cochrane Library** | (root canal OR endodontic) AND (bioceramic sealer OR calcium silicate sealer) AND (resin-based sealer OR epoxy sealer) AND (postoperative pain) | 66 |
| **Scopus** | TITLE-ABS-KEY(("root canal" OR "endodontic treatment") AND ("bioceramic sealer" OR "calcium silicate sealer") AND ("resin-based sealer" OR "epoxy resin sealer") AND ("postoperative pain") AND ("systematic review" OR "meta-analysis")) | 88 |
| **Web of Science** | TS=(("root canal" OR "endodontic treatment") AND ("bioceramic sealer" OR "calcium silicate sealer") AND ("resin-based sealer" OR "epoxy resin sealer") AND ("postoperative pain") AND ("systematic review" OR "meta-analysis")) | 74 |
| **LILACS** | (root canal OR endodontic) AND (bioceramic sealer OR calcium silicate sealer) AND (resin-based sealer OR epoxy sealer) AND (postoperative pain) | 28 |
| **Google Scholar** | “bioceramic sealer” AND “resin-based sealer” AND “root canal treatment” AND “postoperative pain” AND (“systematic review” OR “meta-analysis”) | 46 |
| **Grey Literature (SIGEL, OpenGrey, OpenThesis)** | “root canal” AND (“bioceramic sealer” OR “resin-based sealer”) AND “postoperative pain” AND (“systematic review” OR “meta-analysis”) | 20* |

**Total retrieved before deduplication:** 569 records
**After removal of duplicates and exclusions:** 75 full-texts screened → 7 systematic reviews included.

*Estimated based on grey literature database coverage and overall retrieval proportion.
